# Implications of polyploidy events on the phenotype, microstructure, and proteome of *Paulownia australis*

**DOI:** 10.1371/journal.pone.0172633

**Published:** 2017-03-08

**Authors:** Zhe Wang, Guoqiang Fan, Yanpeng Dong, Xiaoqiao Zhai, Minjie Deng, Zhenli Zhao, Wenshan Liu, Yabing Cao

**Affiliations:** 1 Institute of Paulownia, Henan Agricultural University, Zhengzhou, Henan, P.R. China; 2 College of Forestry, Henan Agricultural University, Zhengzhou, Henan, P.R. China; 3 Forestry Academy of Henan, Zhengzhou, Henan, P.R. China; Universidade de Lisboa Instituto Superior de Agronomia, PORTUGAL

## Abstract

Polyploidy events are believed to be responsible for increasing the size of plant organs and enhancing tolerance to environmental stresses. Autotetraploid *Paulownia australis* plants exhibit superior traits compared with their diploid progenitors. Although some transcriptomics studies have been performed and some relevant genes have been revealed, the molecular and biological mechanisms regulating the predominant characteristics and the effects of polyploidy events on *P*. *australis* remain unknown. In this study, we compared the phenotypes, microstructures, and proteomes of autotetraploid and diploid *P*. *australis* plants. Compared with the diploid plant, the leaves of the autotetraploid plant were longer and wider, and the upper epidermis, lower epidermis, and palisade layer of the leaves were thicker, the leaf spongy parenchyma layer was thinner, the leaf cell size was bigger, and cell number was lower. In the proteome analysis, 3,010 proteins were identified and quantified, including 773 differentially abundant proteins. These results may help to characterize the *P*. *australis* proteome profile. Differentially abundant proteins related to cell division, glutathione metabolism, and the synthesis of cellulose, chlorophyll, and lignin were more abundant in the autotetraploid plants. These results will help to enhance the understanding of variations caused by polyploidy events in *P*. *australis*. The quantitative real-time PCR results provided details regarding the expression patterns of the proteins at mRNA level. We observed a limited correlation between transcript and protein levels. These observations may help to clarify the molecular basis for the predominant autotetraploid characteristics and be useful for plant breeding in the future.

## Introduction

*Paulownia* tree species are native to China, but have been introduced to many other countries for afforestation and land reclamation because of their rapid growth and ability to adapt to poor environmental conditions [1–3]. Additionally, because of their good wood properties, *Paulownia* trees are widely used to make furniture, aircraft, toys, and musical instruments [4]. Collectively, *Paulownia* species have considerable economic and ecological values. Polyploidy events [i.e., whole genome duplications (WGDs)] usually occur in eukaryotes. Generally plant species have polyploid ancestries [5]. Based on parental genome differences, polyploidy is generally classified as either autopolyploid (the duplicated genome from the same species) or allopolyploid (the duplicated genome from the different species). Moreover, plant polyploids generally have large leaves and fruits, as well as other superior traits that make them attractive to breeders; for example, autotetraploid mulberry [6] and allotriploid cucumber [7]. In natural populations, after genomic merging and/or doubling, some genetic combinations may generate new morphological, ecological, physiological, and/or cytological characteristics that may be associated with speciation and biodiversification. The evolutionary success of polyploidy events is indicated by its widespread occurrence, and such events can affect niche differentiation and expand the habitats suitable for a particular plant species. Autopolyploid [8] and allopolyploid [9] are usually able to tolerate adverse environmental conditions better than their progenitors to ensure their survival in heterogeneous conditions. To take advantage of polyploidy events and expand the available *Paulownia* germplasm resources, four autotetraploid *Paulownia* species, including *Paulownia australis*, were synthesized from their diploid relatives using colchicines [10–13]. Autotetraploid *P*. *australis* is more tolerant to salt and drought stresses than diploid *P*. *australis* [14,15].

Changes that are regulated by polyploidy events in plants have become the focus of increasing interest over the past decades. Through the control of circadian-mediated physiological and metabolic pathways, allopolyploid *Arabidopsis* plants improve the synthesis of chlorophyll and starch to increase growth vigor and biomass, and they are larger than their parents [16]. Autopolyploid *Arabidopsis* appear to accumulate more potassium and exhibit greater resistance to the adverse effects of salinity compared with their diploid parents [8]. In *Nicotiana benthamiana*, the artificial octaploid exhibited greater resistance to the adverse effects of drought, cold, and nutrient stress than its allotetraploid parents [17]. In autotetraploid mulberry, the height, leaf size, and fruit size were larger than those of the diploids [6]. Compared with diploids, the cell sizes of autotetraploid *Lolium perenne* and *L*. *multifloru* were bigger because of an increased cell elongation rate, which resulted in increased leaf size [18]. The autotetraploid *Salix viminalis* developed wider leaves with thicker midrib and enlarged palisade parenchyma cells relative to the diploids, and the chlorophyll and carotenoid contents as well as photosynthesis were improved [19]. High-throughput genomic and transcriptomic sequencing techniques have also been used to characterize the changes caused by polyploidy events. Compared to diploid citrus rootstock (*C*. *junos* cv. Ziyang xiangcheng), higher expression level of stress related genes in doubled diploid could be beneficial for its stress tolerance [20]. The differentially expressed genes among Chinese woad (*Isatis indigotica* Fort.) and its autopolyploids were involved mainly in cell growth, response to stress, and photosynthetic pathways [21]. Transcriptome sequencing and comparative analysis of diploid and autotetraploid *P*. *australis* have been preformed, and differentially expressed genes related to carbohydrate and energy metabolism, biosynthesis of cell wall, and stress tolerance have been identified [22]. However, phenotypic changes caused by polyploidy events are not always associated with changes in gene expression. For example, Allario et al found that large changes in anatomy and physiology between diploid lime and its autotetraploid were not associated with large changes in leaf gene expression [23]. Genome- or transcriptome-level expression data may not accurately predict proteome-level changes because regulatory activities such as transcription/post-transcriptional modification, translation/post-translational modification, and protein–protein interactions may influence the composition of the proteome, and it is proteins that play a central role in biological processes. Therefore, protein profile analyses may provide useful functional information that will help to supplement genomic and transcriptomic data.

Two-dimensional gel electrophoresis and isobaric tags for relative and absolute quantitation (iTRAQ) are the main techniques that have been used in proteomic studies. Two-dimensional gel electrophoresis is limited by the under-representation of low-abundance proteins. While, iTRAQ enables the simultaneous identification of many proteins and provides highly sensitive measurements of proteome-level changes. iTRAQ have been used to investigate polyploidy events, such as allopolyploid in *Brassica* species [24] and *Tragopogon mirus* [25], and autopolyploid in *Arabidopsis thaliana* [26]. A proteome study of autotetraploid *Paulownia* ‘Yuza 1’ plants using iTRAQ has been published recently [27]. However, very little is known about the differences in the protein profiles between diploid and autotetraploid *P*. *australis* plants.

The abundant transcript changes between diploid and autotetraploid *P*. *australis* plants have provided some information to characterize the mechanisms involved in plant polyploidization. Knowledge of proteome-level changes resulting from polyploidy events and the associated regulatory activities will help us to understand the mechanisms more throughly. In this study, the morphological, physiological, and microstructural features of *P*. *australis* leaves were analyzed, and a comprehensive iTRAQ-based comparative proteome-level investigation was conducted to study the effects of WGD events. The findings may help to improve our understanding of the molecular basis of polyploidy events.

## Materials and methods

### Plant materials

Plant materials were obtained from the Laboratory of Forest Biotechnology, Henan Agricultural University, Zhengzhou, China. Autotetraploid *P*. *australis* (PA4) was induced from diploid *P*. *australis* (PA2) by colchicine treatment. Their ploidy have been identified by the flow cytometry analysis and chromosome counts [12]. The plants used in this study were tissue cultured seedlings. They were multiplied through *in vitro* plantlet regeneration using leaves from established tissue cultured seedling. The PA2 and PA4 seedlings prepared from the tissue cultures were grown for 30 days at 25 ± 2°C under a 16-h light:8-h dark photoperiod (light intensity: 130 μmol m^−2^ s^−1^). Equal number of leaves from three individual plants were considered as one accession, and at least two biological replicates were used in the experiments. Harvested leaf samples were immediately frozen in liquid nitrogen and stored at −80°C for iTRAQ and quantitative real-time PCR (qRT-PCR).

### Determination of leaf traits

Mature and fully expanded leaves (i.e., second leaf from the apex) were used to measure leaf traits. One leaf from one seedling was considered as one accession. The length and width of at least 10 leaves were measured using a vernier caliper. Chlorophyll content was measured according to a previously described method [28], with three biological replicates for each measurement. Leaf cross-sections were evaluated using scanning electron microscopy. An area approximately 1–2 cm^2^ from the center of each leaf was fixed in 2.5% glutaraldehyde buffered with 0.2 M sodium phosphate buffer (pH 7.2) for scanning electron microscopy. The samples were post-fixed in 1% (w/v) osmium tetroxide for 1 h and then washed three times in the buffer. The samples were dehydrated in a graded alcohol series and then examined in a Quanta 200 environmental scanning electron microscope (FEI, Hillsboro, TX, USA). Three biological replicates were used. The thicknesses of the upper epidermis, palisade layer, spongy parenchyma layer, lower epidermis, and leaf were measured according to the method described by Zhang et al [29]. The cell number and size were determined using Image J software (NIH, Bethesda, MD, USA).

### Protein preparation

The harvested leave samples from PA2 and PA4 were ground to a fine powder in liquid nitrogen, and then treated with lysis buffer (7 M urea, 2 M thiourea, 4% CHAPS, and 40 mM Tris–HCl, pH 8.5) containing 1 mM PMSF and 2 mM EDTA (final concentration). Total protein was extracted and digested according to the method of Tian et al [30].

### Proteome analysis using iTRAQ

The protein profiles of the PA2 and PA4 samples were analyzed using iTRAQ at the Beijing Genomics Institute, Shenzhen, China. The trypsin-digested proteins were labeled using iTRAQ 8-plex kits (Applied Biosystems, Foster City, CA) as follows: PA2-1,113; PA2-2, 114; PA4-1, 117; PA4-2,118, according the method of Qiao et al [31]. The labeled peptides were pooled, dried, redissolved, and fractionated by strong cation exchange (SCX) chromatography using a LC-20AB HPLC pump system (Shimadzu, Kyoto, Japan), as described by Dong et al [27]. The fractionated samples were analyzed by liquid chromatography–electrospray ionization tandem mass spectrometry (LC-ESI-MS/MS) based on the Triple TOF 5600, as described by Qiao et al [31].

Proteins were identified and quantified using the Mascot 2.3.02 search engine (Matrix Science, Boston, MA, USA).The search parameters were as described in a previous study [30].

Proteins were identified using a transcriptome database consisting of 53,230 nonredundant sequences, from sequences that have been submitted to the Short Reads Archive under accession number SRP032321 [22]. To decrease the probability of incorrect protein identifications, only peptides with significance scores (≥ 20) in the 99% confidence interval (i.e., greater than “identity”) of a Mascot probability analysis were considered accurately identified. The identification of each protein was based on at least one unique peptide. To quantify proteins, at least two unique peptides were needed for each protein. The calculated protein ratios were based on weighted averages, and were normalized against the median ratio in Mascot. Proteins with *P* values <0.05 and fold changes >1.2 were considered as differentially abundant proteins (DAPs).

For the functional analyses, the identified proteins were annotated by searching against the Gene Ontology (GO), Clusters of Orthologous Groups (COG), and Kyoto Encyclopedia of Genes and Genomes (KEGG) databases.

### Quantitative real-time PCR analysis of gene expression

Total RNA was extracted from PA2 and PA4 leaves using the TRIzol reagent (Sangon, Shanghai, China). Primers were designed using Beacon Designer (version 7.7) (Premier Biosoft International, Palo Alto, CA, USA) (S1 Table). PCR mixtures with a final volume of 20 μL contained 10 μL SYBR Green PCR mix, 1 μL cDNA, 0.4 μM forward primer, 0.4 μM reverse primer, and 7.0 μL sterile ddH2O. The qRT-PCR was completed using a CFX96 Real-Time System (Bio-Rad) and SYBR Premix Ex Taq II (Takara, Dalian, China). The PCR program was as follows: 95°C for 1 min; 40 cycles of 95°C for 10 s and 55°C for 15 s. Three replicates were analyzed for each gene. Relative gene expression levels were calculated using the 2^−ΔΔCt^ method, and the 18S rRNA of *Paulownia* was chosen as a reference gene for normalization. Student’s *t* test was used to detect differences between PA4 and PA2 at a significance level of *p* = 0.05.

## Results

### Changes on leaf traits after whole genome duplications

**Fig 1:**
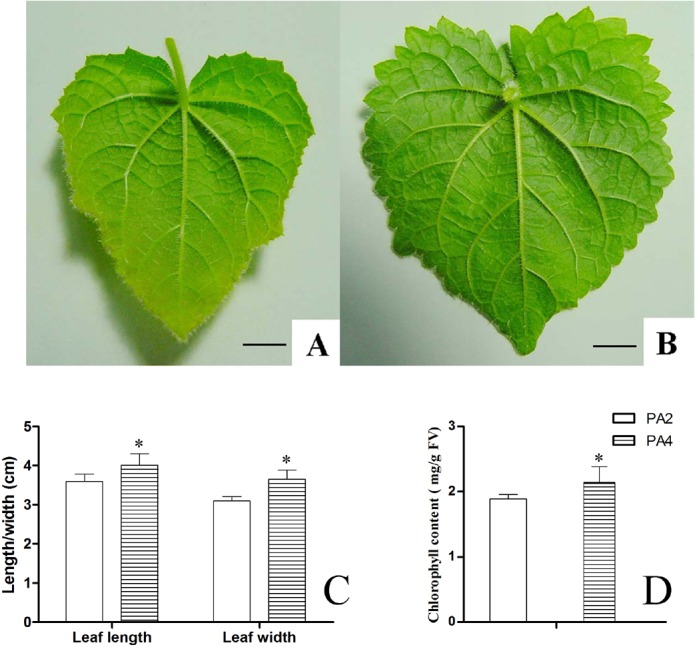
Comparisons of leaves between PA2 and PA4. (A) Leaves of PA2 (B) Leaves of PA4 (C) Length and width of Leaves (D) Chlorophyll content of Leaves. Bar = 1cm. Error bars represent the standard error of the mean. *: Statistically significant differences between PA2 and PA4 (*P*<0.05).

**Fig 2:**
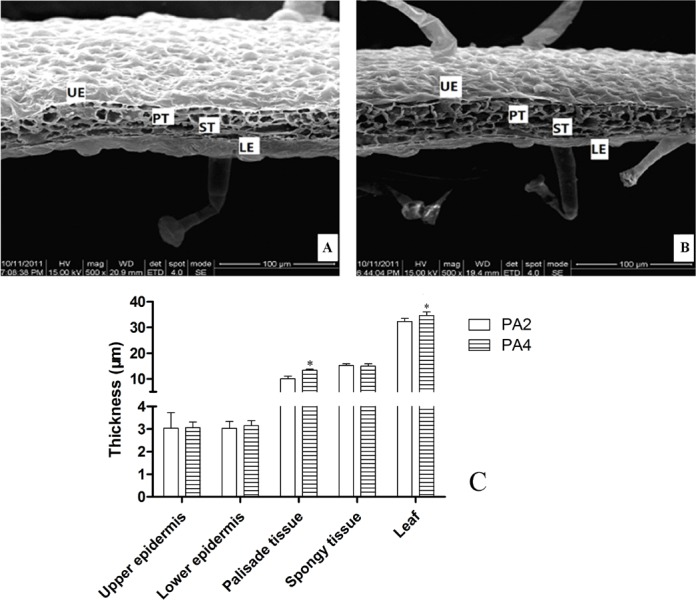
Leaf microstructure of PA2 and PA4. (A) leaf microstructure of PA2 (B) leaf microstructure of PA4 (C) measurents of leaf microstructure. UE, upper epidermal. LE, lower epidermal. PT, palisade tissues.ST, spongy tissues. Bar = 100 μm. The cell numbers in PA2 and PA4 are 176±8 and 140±5, respectively. The cell sizes (mean area) of PA2 and PA4 are 32.60±2.04 um^2^ 37.21±2.04 um^2^. Error bars represent the standard error of the mean. *: Statistically significant differences between PA2 and PA4 (*P*<0.05).

The phenotypic differences among leaves are presented in Fig 1. Compared with PA2 plants, in PA4 plants, leaf length and width were greater; leaf size was bigger. Leaf structures were thicker, including the upper and lower epidermal layers and palisade tissues; but the spongy parenchyma layer was thinner (Fig 2). The cell number was decreased and cell size was increased in PA4 compared with PA2. Chlorophyll content was also higher in PA4 plants than in PA2 plants (Fig 1D), maybe because the thicker palisade tissue of PA4 leaves contained numerous chloroplasts, which are the primary sites for photosynthetic activities. Together, these changes may result in the greater net photosynthetic rate that has been observed in PA4 plants [32].

### iTRAQ data analysis and protein identification

Proteins extracted from PA2 and PA4 leaves were analyzed using an iTRAQ approach. To maximize the number of identified proteins, we limited the peptide matching error during database searches to less than 10 ppm. The distribution of errors between the actual and theoretical relative molecular weights of all matched peptides is presented in S1A Fig. A total of 366,435 spectra were obtained. Based on the analysis with the Mascot search engine, we acquired 21,982 spectra, of which 17,230 were unique spectra. We identified 8,665 peptides, including 7,575 unique peptides, and 3,010 proteins (S1B Fig and S2 Table). The distribution of numbers of peptides, mass and sequence coverage of proteins were shown in S1C, S1D and S1E Fig, respectively. The consistency of the results among the biological replicates implied the proteome-level analyses were reliable (S1F and S1G Fig). The MS/MS raw data generated in this study have been deposited in the ProteomeXchange Consortium database via the PRIDE partner repository (dataset identifier: PXD004237).

To characterize the functions of the 3,010 identified proteins, we first mapped them to the COG database. The proteins were mapped to 23 categories (S2 Fig). In the GO analysis, the identified proteins were classified into 54 groups (S3 Fig). To predict the main metabolic and signal transduction pathways in which the identified proteins involved, we mapped them to KEGG pathways. The proteins were mapped to 120 pathways (S3 Table).

### Analysis of differentially abundant proteins

**Fig 3:**
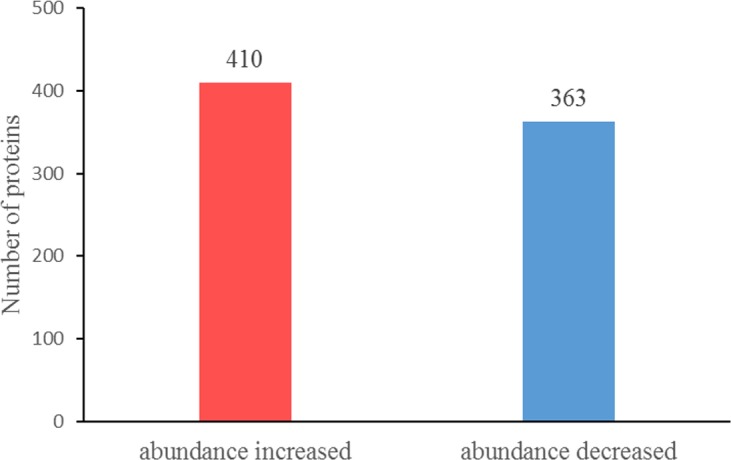
The differentially abundant proteins in PA2 *vs*. PA4.

**Fig 4:**
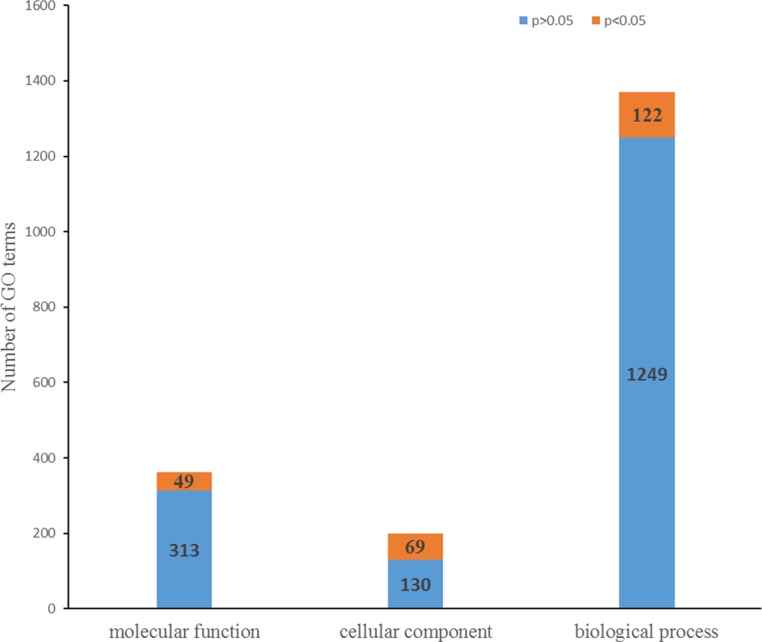
The GO enrichment analyses of the differentially abundant proteins in PA2 *vs*. PA4. *P* value<0.05: GO terms were significantly enriched.

We detected 773 DAPs among the 3,010 identified proteins, including 410 DAPs with higher abundance and 363 DAPs with lower abundance in PA4 compared with PA2 (Fig 3 and S4 Table). We used GO enrichment analyses to determine the main biological functions of the DAPs. Under the biological process category, 122 GO terms were significantly enriched (*P* value<0.05), including “chlorophyll biosynthetic process”, “oxidoreduction coenzyme metabolic process”, and “photosynthesis”. Under the cellular component category, 69 GO terms were significantly enriched, including “chloroplast part”, “plastid part”, and “thylakoid”. Under the molecular function category, 49 GO terms were significantly enriched, including “copper ion binding”, “ion binding”, and “transition metal ion binding” (Fig 4 and S5 Table). The DAPs were also mapped to 101 KEGG metabolic pathways, including the highly enriched “photosynthesis”, “glyoxylate and dicarboxylate metabolism”, “metabolic pathways”, “glutathione metabolism”, and “carbon fixation in photosynthetic organisms” pathways (Table 1). Among the proteins whose abundances were altered by the WGD, the DAPs related to cell division, aerobic respiration, chlorophyll biosynthesis, carbon fixation, and lignin biosynthesis in particular, may provide relevant information regarding the changes induced by the WGD.

**Table 1:** KEGG pathway enrichment analysis of the differentially abundant proteins in PA2 *vs*. PA4.

Pathway	DAPs with pathway annotation	Pathway ID
Photosynthesis*	39 (7.03%)	ko00195
Glyoxylate and dicarboxylate metabolism*	18 (3.24%)	ko00630
Metabolic pathways*	225 (40.54%)	ko01100
Glutathione metabolism*	17 (3.06%)	ko00480
Carbon fixation in photosynthetic organisms*	23 (4.14%)	ko00710
Porphyrin and chlorophyll metabolism	13 (2.34%)	ko00860
Phenylalanine metabolism	11 (1.98%)	ko00360
Valine, leucine and isoleucine biosynthesis	7 (1.26%)	ko00290
Glycolysis / Gluconeogenesis	25 (4.5%)	ko00010
Amino sugar and nucleotide sugar metabolism	18 (3.24%)	ko00520
Tyrosine metabolism	9 (1.62%)	ko00350
Phenylalanine, tyrosine and tryptophan biosynthesis	8 (1.44%)	ko00400
Tropane, piperidine and pyridine alkaloid biosynthesis	4 (0.72%)	ko00960
Fatty acid biosynthesis	7 (1.26%)	ko00061
Photosynthesis—antenna proteins	7 (1.26%)	ko00196
One carbon pool by folate	5 (0.9%)	ko00670
Biosynthesis of secondary metabolites	128 (23.06%)	ko01110
Oxidative phosphorylation	22 (3.96%)	ko00190
Monoterpenoid biosynthesis	2 (0.36%)	ko00902
Other glycan degradation	16 (2.88%)	ko00511
Ascorbate and aldarate metabolism	10 (1.8%)	ko00053
Pyruvate metabolism	20 (3.6%)	ko00620
Isoquinoline alkaloid biosynthesis	5 (0.9%)	ko00950
Fructose and mannose metabolism	11 (1.98%)	ko00051
Citrate cycle (TCA cycle)	14 (2.52%)	ko00020
Glucosinolate biosynthesis	1 (0.18%)	ko00966
Taurine and hypotaurine metabolism	2 (0.36%)	ko00430
C5-Branched dibasic acid metabolism	2 (0.36%)	ko00660
RNA degradation	12 (2.16%)	ko03018
Glycerolipid metabolism	5 (0.9%)	ko00561
Pentose phosphate pathway	11 (1.98%)	ko00030
Cysteine and methionine metabolism	9 (1.62%)	ko00270
Protein export	4 (0.72%)	ko03060
Ribosome	35 (6.31%)	ko03010
Galactose metabolism	12 (2.16%)	ko00052
Arginine and proline metabolism	10 (1.8%)	ko00330
Glycine, serine and threonine metabolism	11 (1.98%)	ko00260
Pantothenate and CoA biosynthesis	3 (0.54%)	ko00770
Glycerophospholipid metabolism	3 (0.54%)	ko00564
Linoleic acid metabolism	1 (0.18%)	ko00591
Nitrogen metabolism	7 (1.26%)	ko00910
mRNA surveillance pathway	11 (1.98%)	ko03015
Alanine, aspartate and glutamate metabolism	8 (1.44%)	ko00250
Proteasome	13 (2.34%)	ko03050
Lysine degradation	2 (0.36%)	ko00310
Limonene and pinene degradation	2 (0.36%)	ko00903
Plant-pathogen interaction	20 (3.6%)	ko04626
Glycosphingolipid biosynthesis—ganglio series	3 (0.54%)	ko00604
Phosphatidylinositol signaling system	3 (0.54%)	ko04070
Nucleotide excision repair	4 (0.72%)	ko03420
Protein processing in endoplasmic reticulum	17 (3.06%)	ko04141
Arachidonic acid metabolism	3 (0.54%)	ko00590
Lysine biosynthesis	2 (0.36%)	ko00300
Valine, leucine and isoleucine degradation	5 (0.9%)	ko00280
Natural killer cell mediated cytotoxicity	1 (0.18%)	ko04650
Brassinosteroid biosynthesis	1 (0.18%)	ko00905
Sulfur relay system	1 (0.18%)	ko04122
Cyanoamino acid metabolism	7 (1.26%)	ko00460
Carotenoid biosynthesis	9 (1.62%)	ko00906
Vitamin B6 metabolism	3 (0.54%)	ko00750
Glycosphingolipid biosynthesis—globo series	2 (0.36%)	ko00603
Butanoate metabolism	4 (0.72%)	ko00650
N-Glycan biosynthesis	1 (0.18%)	ko00510
Sphingolipid metabolism	3 (0.54%)	ko00600
Phagosome	9 (1.62%)	ko04145
Biosynthesis of unsaturated fatty acids	2 (0.36%)	ko01040
Phenylpropanoid biosynthesis	12 (2.16%)	ko00940
Peroxisome	7 (1.26%)	ko04146
alpha-Linolenic acid metabolism	3 (0.54%)	ko00592
Glycosaminoglycan degradation	3 (0.54%)	ko00531
Steroid biosynthesis	1 (0.18%)	ko00100
Cutin, suberine and wax biosynthesis	1 (0.18%)	ko00073
Sulfur metabolism	2 (0.36%)	ko00920
Spliceosome	14 (2.52%)	ko03040
RNA transport	13 (2.34%)	ko03013
Inositol phosphate metabolism	3 (0.54%)	ko00562
Isoflavonoid biosynthesis	1 (0.18%)	ko00943
Riboflavin metabolism	2 (0.36%)	ko00740
beta-Alanine metabolism	2 (0.36%)	ko00410
Propanoate metabolism	4 (0.72%)	ko00640
Endocytosis	5 (0.9%)	ko04144
Aminoacyl-tRNA biosynthesis	5 (0.9%)	ko00970
Zeatin biosynthesis	1 (0.18%)	ko00908
Selenocompound metabolism	1 (0.18%)	ko00450
Histidine metabolism	1 (0.18%)	ko00340
Mismatch repair	1 (0.18%)	ko03430
Tryptophan metabolism	1 (0.18%)	ko00380
Pentose and glucuronate interconversions	4 (0.72%)	ko00040
Terpenoid backbone biosynthesis	3 (0.54%)	ko00900
Homologous recombination	1 (0.18%)	ko03440
Stilbenoid, diarylheptanoid and gingerol biosynthesis	1 (0.18%)	ko00945
Purine metabolism	8 (1.44%)	ko00230
Fatty acid metabolism	2 (0.36%)	ko00071
DNA replication	1 (0.18%)	ko03030
Flavonoid biosynthesis	3 (0.54%)	ko00941
SNARE interactions in vesicular transport	1 (0.18%)	ko04130
Starch and sucrose metabolism	11 (1.98%)	ko00500
Ribosome biogenesis in eukaryotes	1 (0.18%)	ko03008
Pyrimidine metabolism	3 (0.54%)	ko00240
Plant hormone signal transduction	9 (1.62%)	ko04075
Ubiquitin mediated proteolysis	1 (0.18%)	ko04120

*: significantly enriched pathway (*p<0*.*05*).

### Analysis of transcript and protein expression profiles

A combined analysis of the transcriptome and proteome data allows the consistency between transcript and protein profiles to be assessed. The proteins identified by iTRAQ that had transcriptional changes were regarded as correlated with the transcriptome. We investigated the correlation between the profiles of the DAPs identified by the iTRAQ in this study and the mRNA expression profiles from transcriptome-level data from a previous study [22]. We detected 93,272 unigenes that exhibited differences in expression between PA2 and PA4 plants (S6 Table). Among them, we identified 16,490 differentially expressed unigenes (DEUs) using a false discovery rate ≤0.001 and an absolute value of the |log2Ratio| ≥1 as thresholds to determine significant differences in gene expression (S7 Table). A comparison between PA2 and PA4 plants resulted in the identification of 3,010 proteins and 93,272 unigenes. We determined that 3,010 proteins were correlated the transcriptome. Additionally, 1,792 proteins were quantified and correlated. We detected 773 DAPs and 16,490 DEUs, and 129 DAPs was correlated (Table 2).

**Table 2:** Correlation analysis of transcription and proteome.

Group name	Type	Number of Proteins	Number of Genes	Number of Correlations
PA2 *vs*. PA4	Identification	3,010	93,272	3,010
PA2 *vs*. PA4	Quantitation	1,792	93,272	1,792
PA2 *vs*. PA4	Differential Expression	773	16,490	129

Based on the pattern of changes in mRNA and protein levels, four groups proteins were revealed from the quantified proteins: Group I, mRNA and protein levels exhibited the same trends (88 proteins); Group II, mRNA and protein levels exhibited the opposite trends (41 proteins); Group III, mRNA level changed significantly, but the protein level did not (171 proteins); Group IV, protein level changed significantly, but the mRNA level did not (644 proteins) (S8 Table).

**Fig 5:**
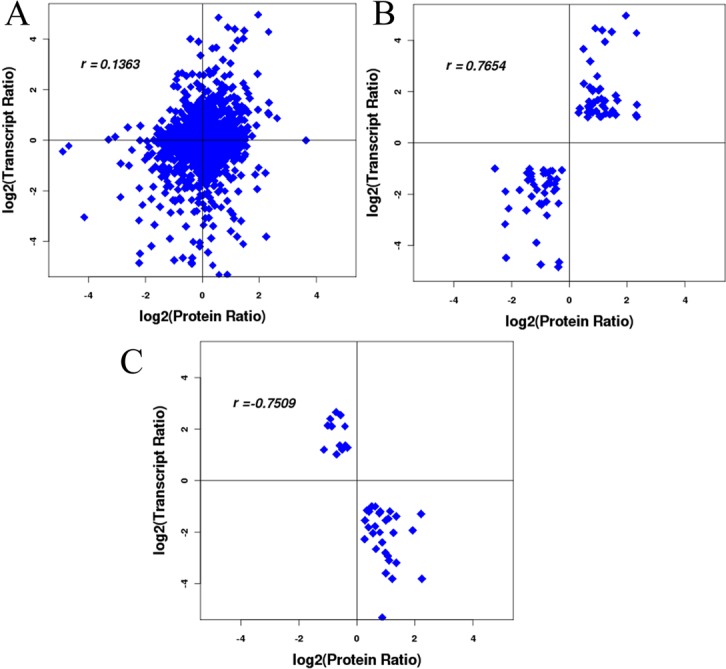
Comparison of expression ratios from transcriptomic and proteomic profiling. (A) The correlation between mRNA and protein when regardless of the direction of the change. (B) The mRNA and protein levels have the same change trends. (C) The mRNA and protein levels have the opposite change trends. Pearson correlations of the comparisons shown in coordinates as r. The protein ratio was from this study. The transcript ratio was reproduced from a previous study [22].

We comprehensively investigated the correlation between protein and mRNA profiles in PA2 and PA4 plants. Poor correlations (Pearson’s *r* value = 0.14) were observed when all quantifiable proteins (1,792) that correlated with a cognate transcript were considered, regardless of the direction of the change (Fig 5A). Correlation between protein and transcript levels of the Group I and Group II members revealed positive (Pearson’s *r* value = 0.77) and negative (Pearson’s *r* value = −0.75) correlations, respectively (Fig 5B and 5C). The discrepancies between the protein and transcript levels may because the mRNA changes did not necessarily lead to similar changes in protein abundance. Alternatively, the proteome-level changes may have been underestimated in this study. Additionally, the identification of DAPs may have been restricted by limitations of the *Paulownia* sequence databases. As noted in other studies, incomplete databases may prevent the detection of all DAPs [24,33]. Although we used a transcriptome database in this study, it lacked sufficient information because the genomes of *Paulownia* species have not been fully sequenced. Thus, the proteins identified using the iTRAQ-based method likely represented only a small part of the proteome. Fully sequencing the genomes of *Paulownia* species to generate more comprehensive databases will likely considerably enhance future proteome-level studies.

### Confirmation of differentially abundant proteins

**Fig 6:**
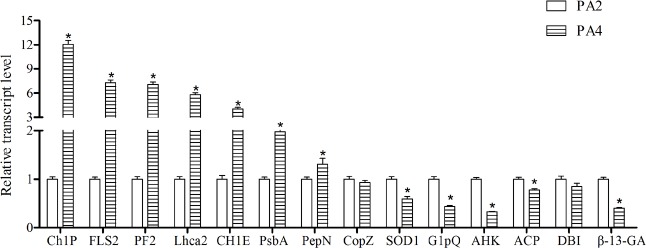
The expression of selected differentially abundant proteins at mRNA level. The 18S rRNA of Paulownia was chosen as an internal reference gene for normalization. ChlP: geranylgeranyl reductase, FLS2: LRR receptor-like serine/threonine-protein kinase FLS2, PF2: plastid fibrillin 2, Lhca2: light-harvesting complex I chlorophyll a/b binding protein 2, ChlE: magnesium-protoporphyrin IX monomethyl ester (oxidative) cyclase, PsbA: photosystem II P680 reaction center D1 protein, PepN: amino peptidase N, CopZ: copper chaperone, SOD1: Cu/Zn superoxide dismutase, GlpQ: glycerophosphoryl diester phosphodiesterase, AHK: arabidopsis histidine kinase, ACP:acid phosphatase, DBI: diazepam-binding inhibitor, β-1,3-GA: beta-1,3-glucanase. Error bars represent the standard error of the mean. *: Statistically significant differences between PA2 and PA4 (*P*<0.05).

To validate the expression change at mRNA level in 14 DAPs, we performed qRT-PCR analysis. The qRT-PCR results revealed that the expression of 13 DAPs at mRNA level were consistent with their protein expression (Fig 6). The discrepancy between the qRT-PCR and iTRAQ results for another DAP may be explained by post-transcriptional and/or post-translational regulatory processes.

## Discussion

Polyploidy events are crucial for plant evolution, and are associated with the origins of most angiosperms and ferns [34,35]. Polyploidy is believed to increase plant size and enhance adaptations to environmental stresses [36,37].

### Variations of leaf parameters

In this study, we determined that leaf length and width increased following WGDs, and speculated that this may be caused by the variations of cell division and expansion [38]. Leaf size is controlled by an organ-wide integration system with two main factors: cell number and size. Increases in cell size can result from a decrease in cell number and *vice versa* [39]. In our study, compared with PA2, PA4 has less cell number and bigger cell size. Sugiyama [18] found the bigger leaf in autotetraploid *Lolium perenne* and *L*. *multifloru* compared with diploid was a result of increased cell size, which is similar to the finding in this study. In mulberry, the cell size of autotetraploid was larger than in diploid [6]. Autotetraploid *Salix viminalis* has wider leaves and enlarged cells [19]. It appears that autopolyploidy can result in increased cell and leaf sizes. PA4 had thicker upper and lower epidermis than PA2. Among diploid, pentaploid, and hexaploid *Betula papyrifera*, the polyploids had thicker upper and lower epidermis [40], which is similar with our results. The amount of light absorbed by a leaf is related to its chlorophyll content [41]. In PA4, the chlorophyll content was higher than in PA2, suggesting that PA4 could absorb more light.

Besides improvements in agricultural productivity and efficiency (biomass) through the phenotypic variations caused by polyploidization, stress tolerance will also become higher [20]. Leaf is highly responsive to environmental conditions, therefore, its microstructures may reflect the adaptability of plants to environmental conditions [42]. The olive Chemlali cultivar exhibited more tolerance to water stress than the Chétoui cultivar, and its palisade was thicker [43]. The diploid *Betula papyrifer*a, with thinner upper and lower epidermis, was more sensitive to water deficit than the polyploids [40]. The leaf microstructure observed in the PA2 and PA4 plants may explain the higher tolerance to stresses of PA4 compared with PA2.

### Differentially abundant proteins related to leaf trait variability

**Fig 7:**
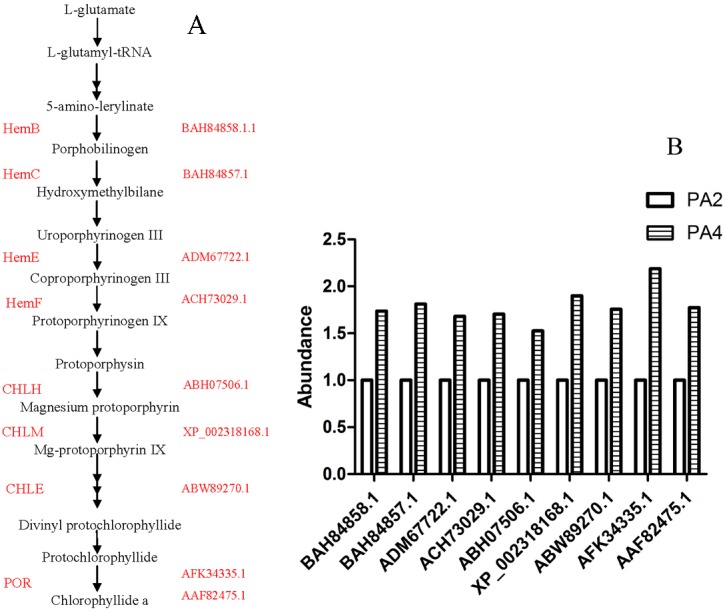
Chlorophyll biosynthesis-associated differentially abundant proteins. (A) Chlorophyll synthetic pathway in *P*.*australis*. (B) The abundance of differentially abundant proteins. The abundance of proteins in PA2 was 1. HemB: porphobilinogen synthase, HemC: hydroxymethylbilane synthase, HemE: uroporphyrinogen decarboxylase, HemF: coproporphyrinogen III oxidase, CHLH: magnesium chelatase subunit I, CHLM: magnesium-protoporphyrin O-methyltransferase, CHLE: magnesium-protoporphyrin IX monomethyl ester cyclase, POR: magnesium chelatase subunit protochlorophyllide reductases.

**Fig 8:**
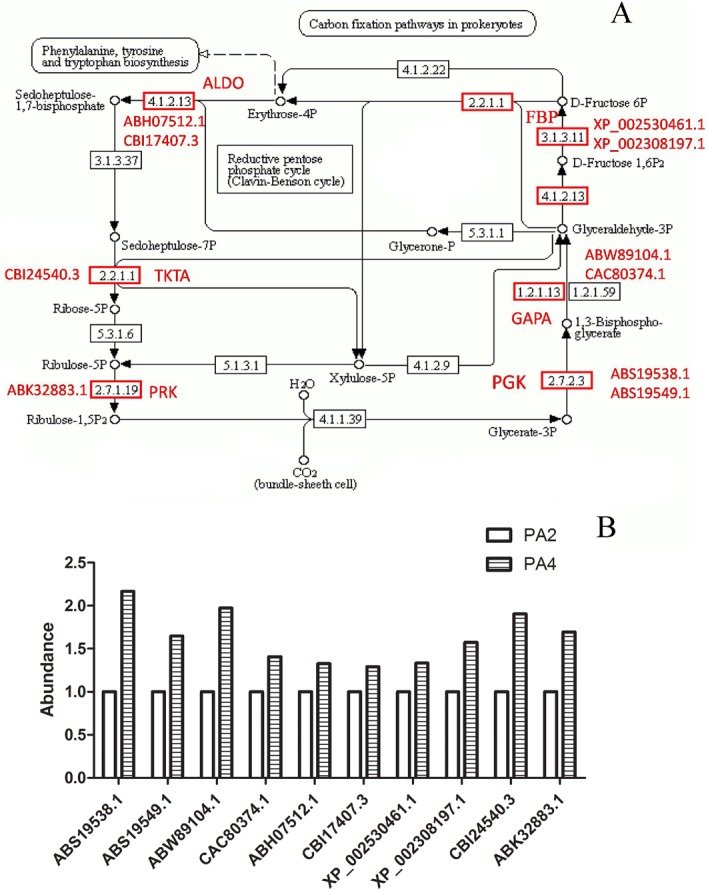
Photosynthesis-associated differentially abundant proteins. (A) Carbon fixation pathway in *P*.*australis*. (B) The abundance of differentially abundant proteins. The abundance of proteins in PA2 was 1. PGK: phosphoglycerate kinase. GAPA: glyceraldehyde-3-phosphate dehydrogenase. ALDO: fructose-bisphosphate aldolase, class I. FBP: fructose-1,6-bisphosphatase I. TKTA: transketolase. PRK: phosphoribulokinase.

In this study, we determined that leaf length and width, and leaf size increased following WGDs. Cell division is one of the fundamental processes for plant organ growth and development [18]. Four DAPs (XP_002282146.1, AFJ42570.1, BAD45447.1, and NP_001183829.1) related to cell division were more abundant in PA4 plants than in PA2 plants. Larger cells may promote cell wall synthesis. Cellulose is a major component of plant cell walls, and two DAPs (CBI16310.3 and AFK34932.1) associated with cellulose biosynthesis were more abundant in PA4 than in PA2. Increases in leaf size may occur as a result of an increase in the cell elongation rate and/or by a prolonged cell elongation period, both of which require energy and materials. The F-type ATPases catalyze ATP synthesis, and the two F-type H^+^-transporting ATPase subunits (A29394 and AAQ84325.1) that were more abundant in PA4 plants than in PA2 plants might be responsible for supplying the energy required to increase leaf length. Although there was no direct relationship between leaf size and chlorophyll content, the thicker palisade tissues were associated with elevated chlorophyll content. We identified eight DAPs involved in chlorophyll biosynthesis that were more abundant in PA4 than in PA2 (Fig 7). The variability in abundance of these DAPs may help to clarify the molecular basis of elevated chlorophyll content in PA4. We also identified 10 DAPs related to carbon fixation that were more abundant in PA4 than in PA2 (Fig 8). This suggested increased chlorophyll content and photosynthetic activities, which is consistent with the results of a previous study [32]. Chlorophyll content may be an indicator of photosynthetic capacity.

### Differentially abundant proteins related to stress resistance

**Fig 9:**
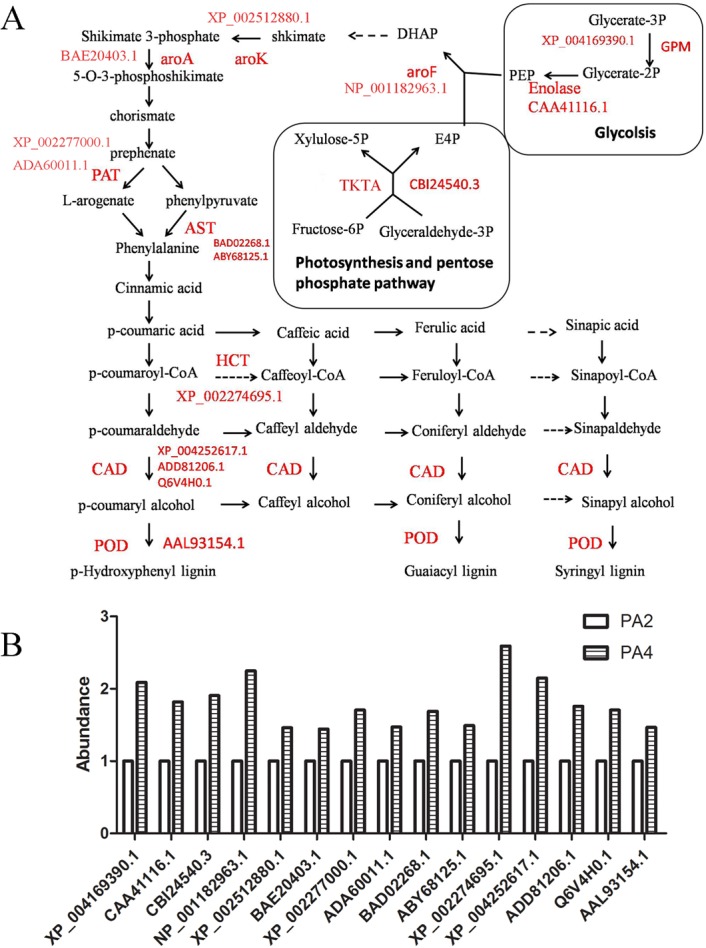
Lignin biosynthesis-associated differentially abundant proteins. (A) A diagram shows the intermediates and enzymes involved in lignin biosynthesis pathway in *P*.*australis*. (B) The abundance of differentially abundant proteins. The abundance of proteins in PA2 was 1. TKTA: transketolase, GPM: phosphoglycerate mutase, aroF: 3-deoxy-7-phosphoheptulonate synthase, aroK: shikimate kinase, aroA: 5-enol-pyruvylshikimate-3-phosphate synthase, PAT: glutamate/aspartate-prephenate aminotransferase, AST: aspartate transaminase, HCT: shikimate/quinate hydroxyl cinnamoyl transferase, CAD: cinnamyl alcohol dehydrogenase, POD: peroxidase.

Polyploids usually have better fitness than their progenitors [17]. Lignin deposition generally occurs when cell growth is complete and the cell wall is undergoing secondary thickening via lignification. Among its many and varied biological roles, lignin is important for plant adaptations to diverse environmental conditions [44,45]. Determining the changes in abundance of lignin-related proteins may provide relevant information regarding how polyploidy events increase plant adaptability. The DAPs related to lignin synthesis were accumulated after the WGD, including, for example, transketolase (TKTA), phosphoglycerate mutase (GPM), enolase, 3-deoxy-7-phosphoheptulonate synthase (aroF), shikimate kinase (aroK), 5-enol-pyruvylshikimate-3-phosphate synthase (aroA), glutamate/aspartate-prephenate aminotransferase (PAT), aspartate transaminase (AST), shikimate/quinate hydroxyl cinnamoyl transferase (HCT), cinnamyl alcohol dehydrogenase (CAD), and peroxidase (POD) (Fig 9).

Lignin is produced by the oxidative coupling of three monolignols which are synthesized from phenylalanine via the phenylpropanoid pathway. Chorismate originates from the shikimate pathway, and is a common intermediate in the biosynthetic pathways for phenylalanine and other aromatic amino acids. Chorismate is initially synthesized from D-erythrose 4-phosphate (E4P) and phosphoenolpyruvate (PEP), which are produced by the pentose phosphate pathway and glycolysis, respectively.

The immediate substrates of the shikimate pathway are E4P and PEP [46]. Additionally, TKTA catalyzes reactions in the Calvin cycle and oxidative pentose phosphate pathway to produce E4P. Henkes et al. observed that down-regulated TKTA activity results in decreased abundance of aromatic amino acids and lignin [47]. The carbon flux towards the shikimate pathway and phenylpropanoid metabolism is restricted by the supply of E4P. GPM catalyzes the conversion of 3-phosphoglycerate to 2-phosphoglycerate, and enolase catalyzes the production of PEP from 2-phosphoglycerate. TKTA, GPM, and enolase were more abundant in PA4 than in PA2, and may increase the carbon resources available for the shikimate pathway.

3-Deoxy-7-phosphoheptulonate (DHAP) synthase regulates the amount of carbon entering the pathway and catalyzes the conversion of E4P and PEP to DHAP [48]. AroK catalyzes the conversion of shikimate to shikimate-3-phosphate, with ATP as a co-substrate [49]. The two substrates for aroA are PEP and shikimate-3-phosphate, which are converted to phosphate and 5-enol-pyruvylshikimate-3-phosphate [50]. There are two routes for the post-chorismate branch of the pathway leading to phenylalanine synthesis (i.e., phenylpyruvate and arogenate routes). AST is part of the phenylpyruvate route, while PAT is involved in the arogenate route [51]. Both of these enzymes were more abundant in PA4 than in PA2, and may lead to increased phenylalanine levels.

Lignin is derived from phenylalanine. The monolignols produce the p-hydroxyphenyl, guaiacyl, and syringyl phenylpropanoid units of the lignin polymer. The HCT enzyme represents a key metabolic entry point for the synthesis of the most important lignin monomers. CAD catalyzes the reduction of cinnamyl aldehydes to the corresponding cinnamyl alcohols, which are the monomeric precursors of lignin [52]. The last step in lignin biosynthesis involves the polymerization of cinnamyl alcohols, which is affected by POD activities. In this study, we found that HCT, CAD, and POD were more abundant in PA4 plants than in PA2 plants. This may result in increased lignin production to strengthen cell walls and protect plants from environmental stresses. After cellulose, lignin is the most abundant terrestrial biopolymer, comprising approximately 30% of the organic carbon in the biosphere. Furthermore, the increased lignin content of PA4 plants may lead to enhanced carbon fixation, and ultimately increased biomass.

In plants, glutathione (GSH) is crucial for biotic and abiotic stress tolerance. We identified some DUPs related to GSH metabolism. Up-regulating GSH synthesis is likely an intrinsic plant response to stress [53]. Glutamate–cysteine ligase (gshA) may promote the synthesis of GSH [54]. GshA is an important component of the glutathione–ascorbate cycle that decreases hydrogen peroxide levels. In response to stress conditions, PA4 plants may increase gshA abundance to enhance their tolerance to environmental stresses. Abiotic stresses, such as drought and high salinity, damage plants via exposure to oxidative stress, which may induce the rapid generation of reactive oxygen species (ROS). Eliminating ROS improves plant growth. Glycine betaine (GB) is important for maintaining the enzymatic activities responsible for eliminating or detoxifying ROS [55,56]. Accumulating GB may enable plants to continue to grow under drought or salt stress conditions. Additionally, GB is synthesized from glycine, which is derived from serine. Glycine hydroxymethyltransferase (glyA) may promote the synthesis of glycine from serine. Increased abundance of glyA (XP_004253097.1, AFA36570.1, and CBI17302.3) may be conducive for the accumulation of GB. The adverse effects of ROS are also mitigated by superoxide dismutase. Previous studies revealed that the superoxide dismutase content was higher in PA4 leaves than in PA2 leaves [14,15]. Damage caused by ROS is amplified by the accumulation of toxic degradation products (e.g., aldehydes) arising from reactions between ROS and lipids or proteins. Aldehyde dehydrogenases eliminate toxic aldehydes [57], and in *A*. *thaliana*, they were shown to influence salinity tolerance [58]. In this study, we observed that aldehyde dehydrogenases (EMJ14942.1 and ACM89738.1) were accumulated in PA4 plants, possibly contributing to enhanced tolerance to environmental stresses.

### Correlation between transcript and protein

The proteome and transcriptome reflect the situation of gene expression at two different levels. Combining these two omics data can help to reveal more complete expression information for an organism. Correlation between proteome and transcriptome data may reveal the relation between protein abundance and mRNA transcript levels. In yeast, the correlation between transcript expression and protein abundance was good [59], and in *Tragopogon mirus* [25], *Brassica napus* [60], and *A*. *thaliana* [26,61], limited correlations were found. Thus, it may be considered that low correlation between mRNA expression and protein abundance is common. In this study, we found non-significant correlation between proteomic and transcriptomic changes, suggesting the important role of post-transcriptional regulation and translational modification changes of proteins during the polyploidization process in *P*. *australis*. To comprehensively investigate the correlation, we clustered the quantified proteins into four groups. The information from the correlated DAPs and DEUs that had same change trends (Group I) may verify and explain some important processes; for example, the response to environmental stress in fission yeast [59]. The correlated DAPs and DEUs that had opposite change trends (Group II) may explain some phenomenon that could not explained by only proteome or transcriptome data; for example, the plant dormancy process [62]. Groups I and II together contained 129 proteins. Groups III and IV, which contained significantly changed mRNAs associated with unchanged proteins or significantly changed proteins correlated to unchanged mRNAs, contained 815 proteins. The differences in these numbers may result from the definition of DEUs and DAPs. Nonetheless, in many studies, in order to understand the effects of polyploidization, integrated analysis of transcriptome and proteome data is essential.

## Conclusions

To characterize the molecular and biological mechanisms that regulate the effects of polyploidy events on *P*. *australis*, we compared the leaf parameters and proteomes of autotetraploid and diploid *P*. *australis*. The identification of DAPs related to cell division, GSH metabolism, and the synthesis of cellulose, chlorophyll, and lignin may help to characterize the differences between the diploid and autotetraploid *P*. *australis* plants, and clarify the mechanisms involved in regulating polyploidy events. Our results provide an overview of the changes caused by WGDs in *P*. *australis* and may be useful for breeding new *Paulownia* species and expanding the available germplasm resources.

## Supporting information

S1 Fig Overview of the iTRAQ data. (A) Error distribution of spectra match quality. (B) Proteome identification. (C) Number of peptides that match proteins. (D) Protein relative molecular mass. (E) Coverage of the proteins by the identified peptides. (F) The repeatability of two replicates of PA2 and (G) The repeatability of two replicates of PA4. 1 and 2 represent two biological replicates of samples. The ratios of protein abundances for each protein in each comparison between biological replicates were calculated, and the “delta, error” in the absciss are presents the difference from the expected ratio of 1.
(TIF)

S2 Fig Clusters of Orthologous Groups classification of protein in leaves of *P. australis*. 1768 proteins were divided into 23 specific categories.
(TIF)

S3 Fig Gene Ontology (GO) classification of protein in leaves of *P. australis*. 2738 proteins were categorized into 51 function groups.
(TIF)

S1 Table Primer sequence of corresponding unigenes of selected differentially abundant proteins. (XLSX)

S2 Table Detail of the all identified proteins. (XLSX)

S3 Table KEGG annotation of the identified proteins. (XLSX)

S4 Table Differentially abundant proteins in PA2 *vs.* PA4. (XLSX)

S5 Table GO enrichement of the differentially abundant proteins in PA2 *vs.* PA4. (XLSX)

S6 Table Differentially expressed unigenes from previously transcriptome study. (XLSX)

S7 Table Significant differentially expressed unigenes from previously transcriptome study. (XLSX)

S8 Table Analysis of the correlation between mRNA and protein abundance changes. (XLSX)
