# Supplementary figures and images for: Implications of polyploidy events on the phenotype, microstructure, and proteome of Paulownia australis
Source: PLoS One. 2017 Mar 8;12(3):e0172633. doi: 10.1371/journal.pone.0172633 (PMC5342211; doi:10.1371/journal.pone.0172633)

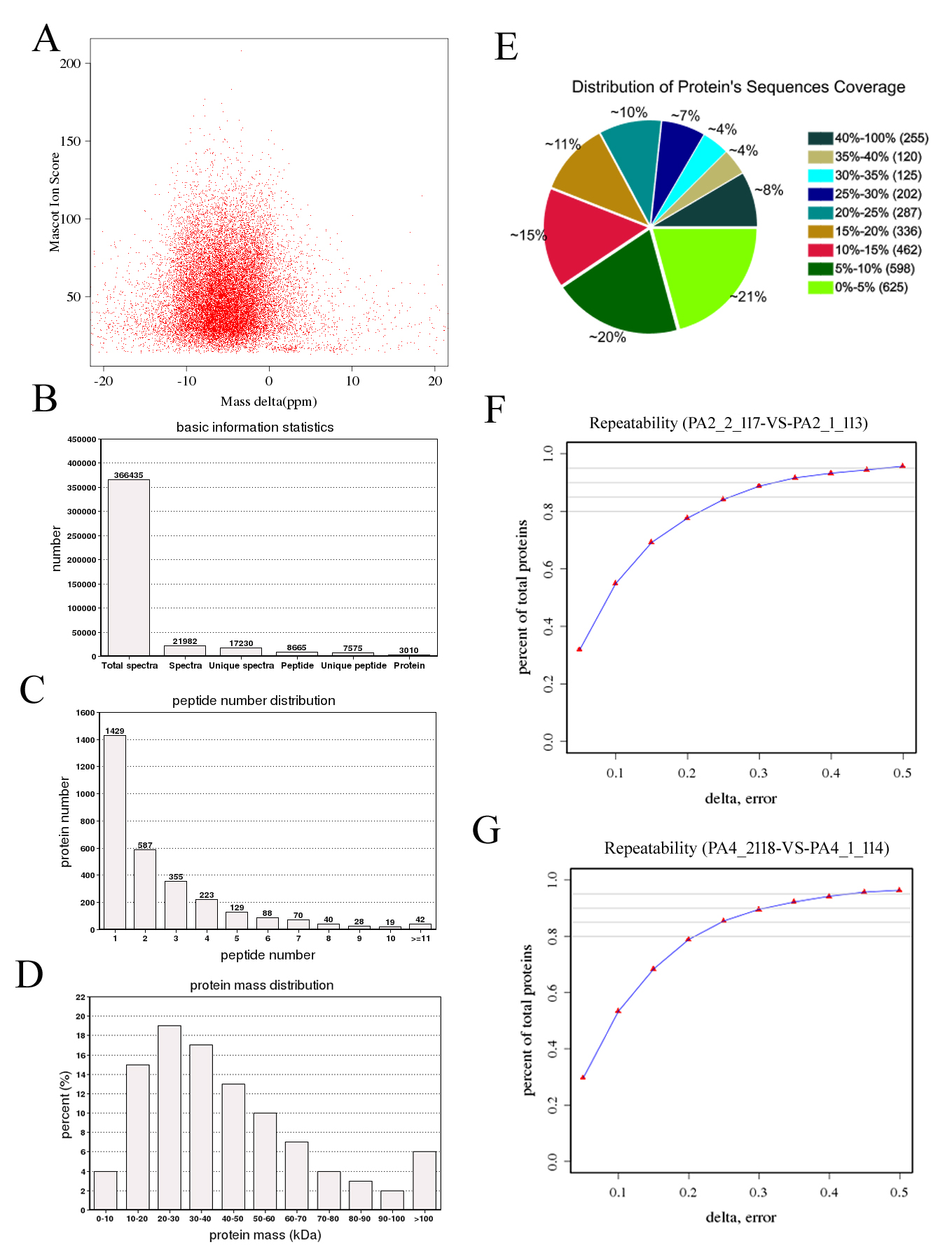

Supplement: S1 Fig — (A) Error distribution of spectra match quality. (B) Proteome identification. (C) Number of peptides that match proteins. (D) Protein relative molecular mass. (E) Coverage of the proteins by the identified peptides. (F) The repeatability of two replicates of PA2 and (G) The repeatability of two replicates of PA4. 1 and 2 represent two biological replicates of samples. The ratios of protein abundances for each protein in each comparison between biological replicates were calculated, and the “delta, error” in the absciss are presents the difference from the expected ratio of 1. (TIF) [file pone.0172633.s001.tif]

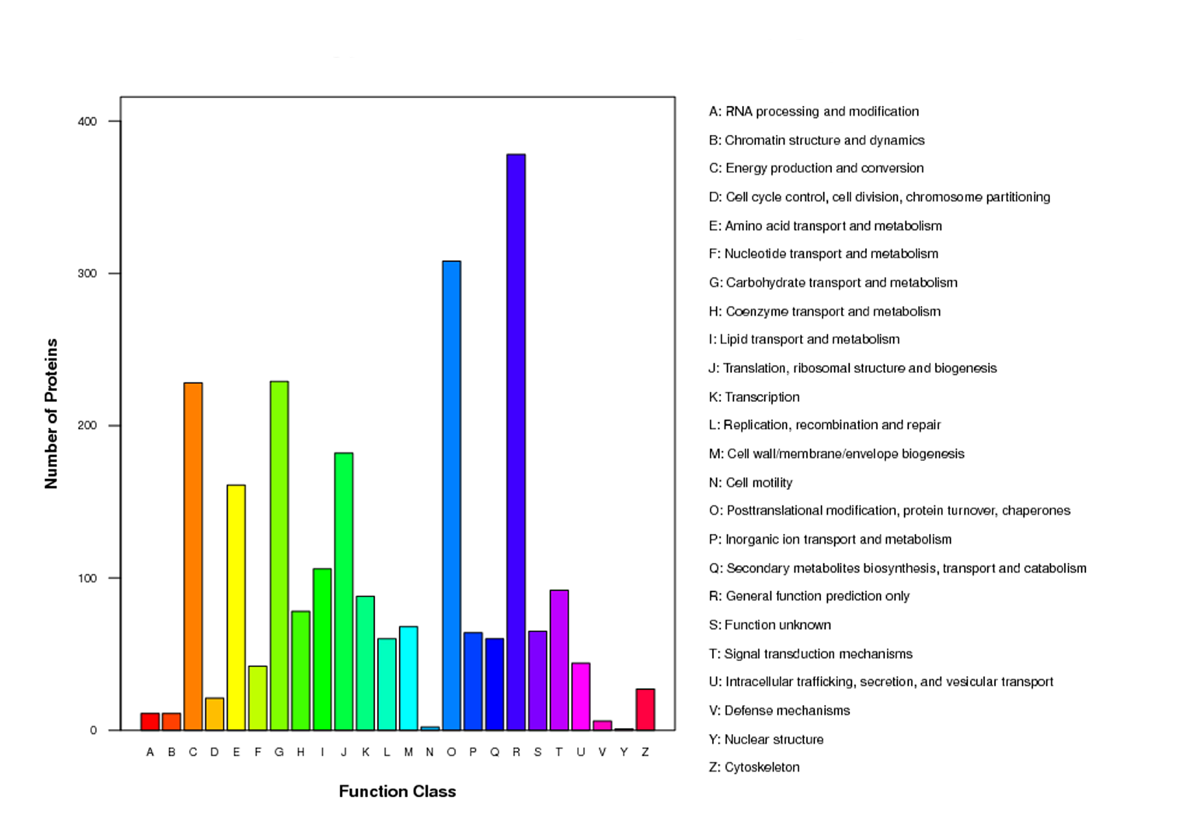

Supplement: S2 Fig — 1768 proteins were divided into 23 specific categories. (TIF) [file pone.0172633.s002.tif]

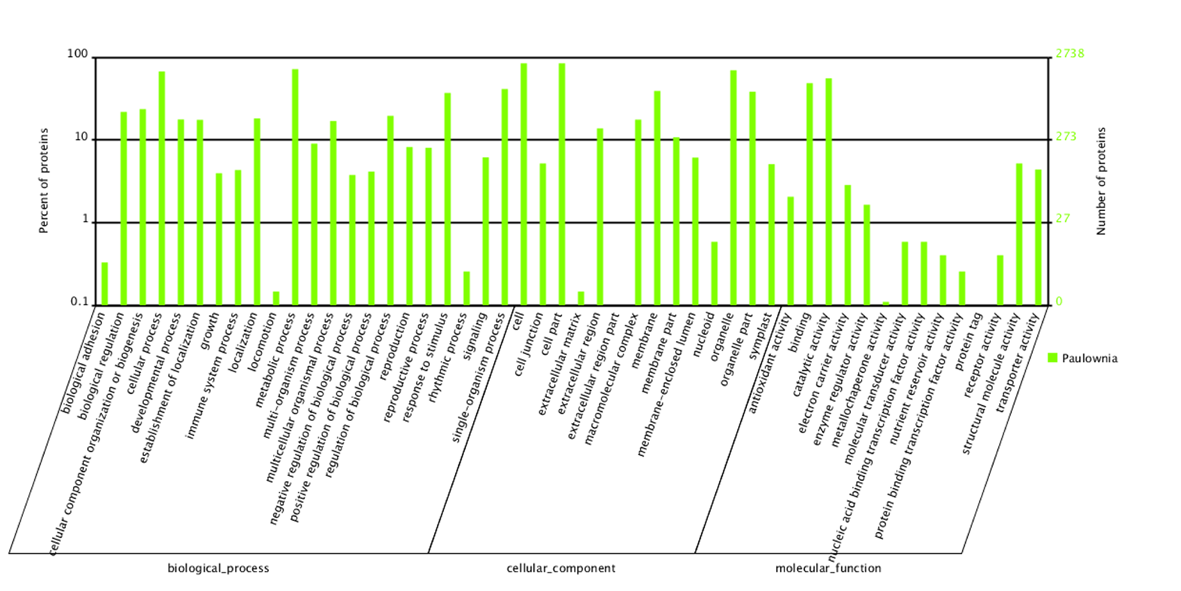

Supplement: S3 Fig — 2738 proteins were categorized into 51 function groups. (TIF) [file pone.0172633.s003.tif]
